# Supplementary material for: Mammographic Density Changes over Time and Breast Cancer Risk: A Systematic Review and Meta-Analysis
Source: Cancers (Basel). 2021 Sep 25;13(19):4805. doi: 10.3390/cancers13194805 (PMC8507818; doi:10.3390/cancers13194805)
Supplement: Supplementary file 1 [file cancers-13-04805-s001.zip › cancers-1363246-supplementary.pdf]

Supplementary material

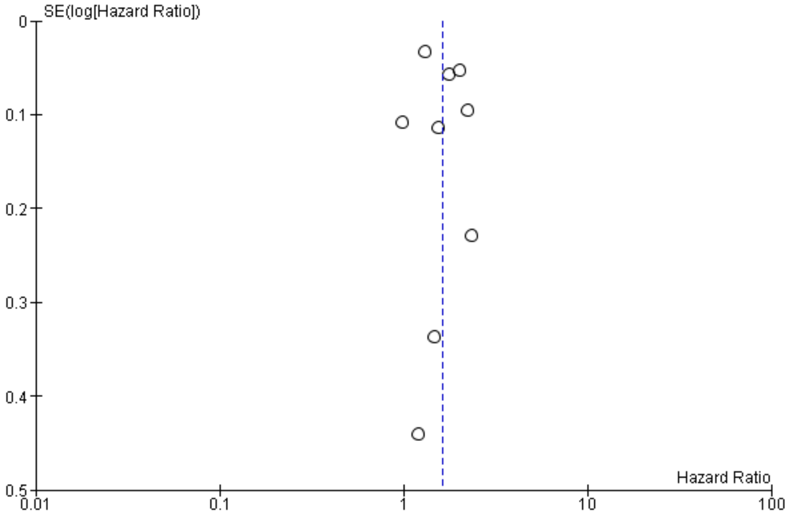

Figure S1 Funnel plot for pooled analysis of breast cancer risk in relation to in-creased breast density over time (cohort studies)

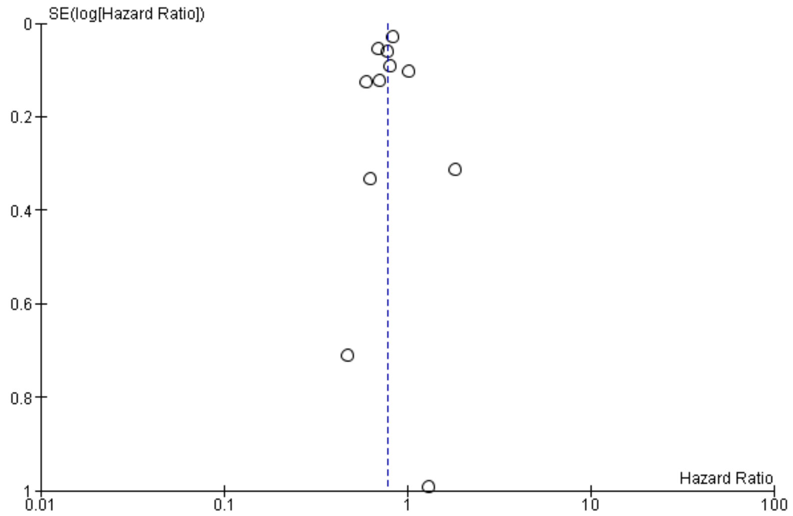

Figure S2. Funnel plot for pooled analysis of breast cancer risk in relation to de-creased breast density over time (cohort studies)

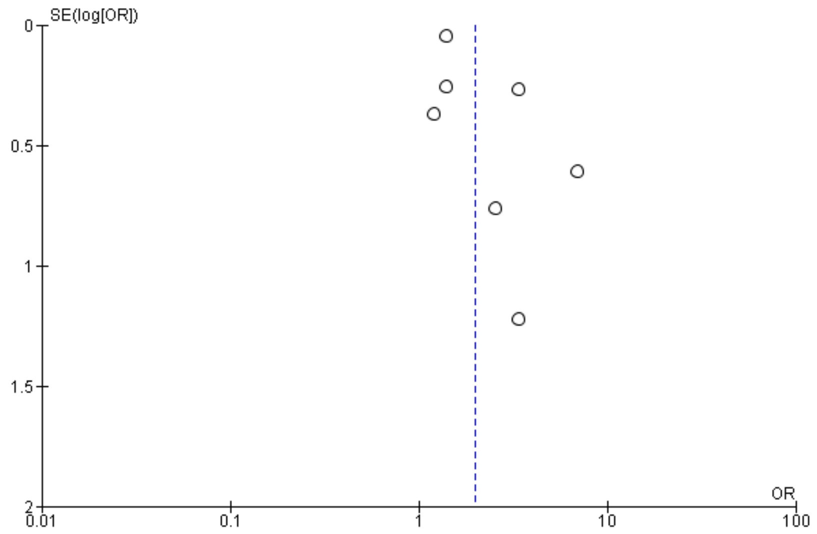

Figure S3. Funnel plot for pooled analysis of breast cancer risk in relation to in-creased breast density over time (studies presenting Odds Ratios)

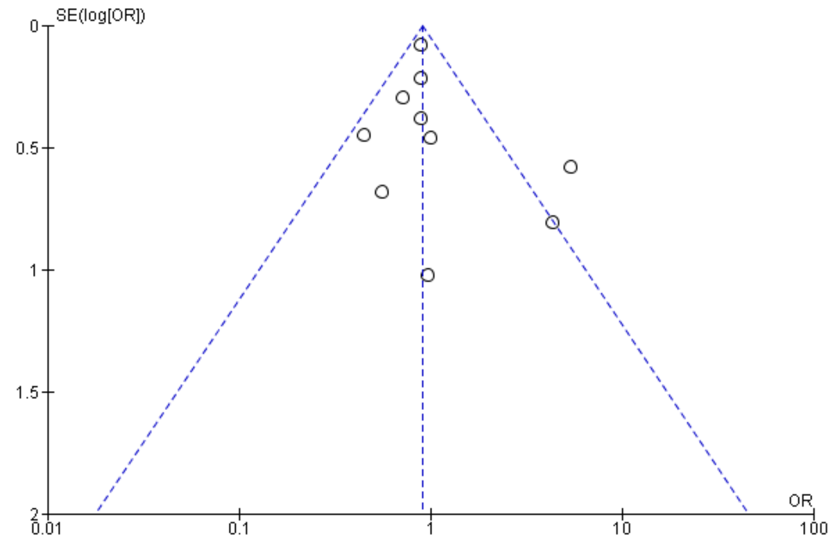

Figure S4. Funnel plot for pooled analysis of breast cancer risk in relation to de-creased breast density over time (studies presenting Odds Ratios)
